# Natal Versus Breeding Dispersal in Arctic Terns ( *Sterna paradisaea* ): A Banding Study From Denmark (1931–2005)

**DOI:** 10.1002/ece3.74372

**Published:** 2026-09-24

**Authors:** Haiyu Li, Anders Pape Møller, Shengyu Wang, Céline Albert, Jacqui A. Shykoff, Ivan Jarić

**Affiliations:** ^1^ School of Biological Sciences Monash University Clayton Australia; ^2^ Université Paris‐Saclay, CNRS, AgroParisTech, Écologie Société Évolution Gif‐sur‐Yvette France; ^3^ Norwegian Institute for Nature Research Trondheim Norway; ^4^ Biology Centre of the Czech Academy of Sciences Institute of Hydrobiology České Budějovice Czech Republic

**Keywords:** habitat selection, migration, nesting, regional connectivity characteristics, *Sterna paradisaea*

## Abstract

The dispersal of migratory birds is a crucial process that directly influences population viability, genetic connectivity and adaptive potential in changing environments. Using banding data spanning 75 years (1931–2005) for Arctic Terns ( *Sterna paradisaea* ) in Denmark, we identified 200 breeding dispersal and 890 natal dispersal events. We characterised their spatial and temporal patterns and compared the differences between natal and breeding dispersal. We found limited dispersal distances and restricted directional preferences, as well as a low trans‐regional exchange rate during breeding dispersal. In contrast, natal dispersal exhibited longer distances, more diverse directional preferences and a higher frequency of cross‐regional movement events. This study offers a valuable long‐term dataset and a descriptive foundation for future research and conservation of these migratory birds.

## Graphical abstract

We summarise Denmark's long historical dispersal events, understanding their directional, distance and regional flow characteristics. Comparing natal dispersals and breeding dispersals, provides insights for further research.

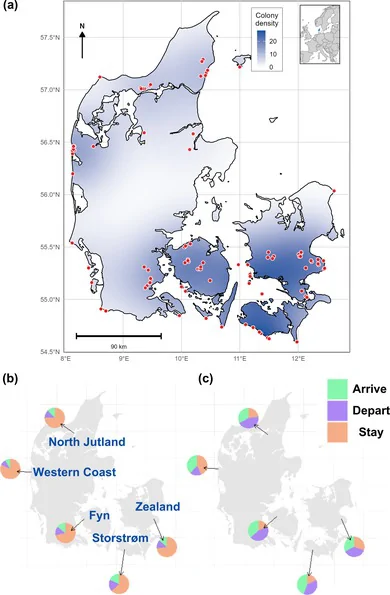

## 1 Introduction

Dispersal is a key ecological process that can affect population connectivity, gene flow and the long‐term viability of long‐migratory species (Bowler and Benton 2005). Both juveniles and adults can undergo dispersal, including natal dispersal (i.e., a shift from the natal colony the first breeding colony for juveniles) and breeding dispersal (i.e., a shift to a different breeding colony between breeding seasons for adults) (Greenwood and Harvey 1982). These events are determined by both intrinsic and extrinsic factors (Paradis et al. 1998), which differ between juveniles and adults, resulting in their different dispersal patterns. For example, in piping plover ( *Charadrius melodus* ), natal dispersal spans substantially longer distances (Swift et al. 2021).

Arctic tern ( *Sterna paradisaea* ) is a long‐distance migratory seabird that breeds in Arctic and sub‐Arctic regions, and overwinters in Antarctic waters (Fijn et al. 2013). This species is highly dependent on coastal ecosystems for breeding (Hatch et al. 2020). Although it migrates between polar regions each year, it has strong site fidelity at the breeding colony (> 90% return rate in North America; Devlin et al. 2008; 71%–88% apparent natal philopatry in a UK metapopulation; Redfern et al. 2026). Due to high site fidelity, dispersal events, which represent changes between colonies, are rare in documented long‐term banding records (O'Hanlon et al. 2023). However, understanding these events is crucial for conservation, as they create inter‐population gene flow that drives adaptation to changing environments (Gaya et al. 2024).

Over the past century, seabirds have faced pressure from pollutant emissions that accumulate in coastal regions and marine food webs, reducing their reproductive success and survival (Rhind 2009; Macdonald et al. 2005; Dias et al. 2019). These pressures are compounded by broader anthropogenic impacts, including intensifying commercial fishing and carbon emissions, which are reshaping coastal ecosystems and food availability worldwide, including in the Arctic and sub‐Arctic regions (McKinney and Lockwood 1999; Trenberth 2018). Arctic terns are exposed to these pressures across their distribution range, and their breeding populations are declining (BirdLife International 2018). Given these threats and the ongoing decline of Arctic terns, understanding dispersal patterns is key for effective conservation planning, because dispersal determines the actual adaptive potential of a population to changing habitat conditions (Thurman et al. 2020; Redfern et al. 2026). Quantifying their dispersal locations, distances and frequencies can help develop effective and targeted conservation strategies and provide insights for the conservation of other long‐distance migratory birds in high‐latitude regions (Wakefield et al. 2017).

Denmark has experienced a strong population decline of this species. The national breeding population of Denmark has fallen from 8000 to 10,000 pairs in the 1970s–1990s to about 2150 pairs in 2023, and has decreased by more than half since 2006 (NOVANA monitoring programme, https://novana.au.dk/fugle; Nielsen et al. 2024). The breeding colonies of Arctic terns in Denmark are regionally clustered along the coastlines of various archipelagos and the Jutland Peninsula, characterised by both regional dispersal and interregional communication within a single monitored population. A banding dataset has tracked Arctic terns' breeding colonies since 1931, with the data previously used to assess potential effects of climate change (Møller et al. 2006) and agricultural fertilisers (Møller et al. 2007). However, no research focused on the spatial and temporal patterns of dispersal events themselves.

Here, we examine the natal and breeding dispersal patterns of Arctic terns in Denmark using a 75‐year banding dataset comprising 15,015 records from 242 breeding colonies. Specifically, we compare the proportion of individuals undertaking dispersal, dispersal distance and dispersal direction between natal and breeding dispersal, assess how these vary over time and evaluate the proportion of within‐ versus cross‐region movements among five regions (Northern Jutland, Western Coast, Copenhagen Metropolitan, Funen and Storstrøm). Adults tend to return to a colony whose quality, neighbours and pair bond they have already established, whereas first‐time breeders settle with none of these (Greenwood and Harvey 1982; Chalfoun and Schmidt 2012). We therefore hypothesise that breeding dispersal is more spatially constrained than natal dispersal, characterised by a lower occurrence rate, shorter distances, increased directional bias and lower rates of inter‐regional exchange. This study has important significance for migratory bird conservation: breeding dispersal determines whether established adults can leave a deteriorating colony, whereas natal dispersal determines how colonies remain connected (Runge et al. 2014; Marra et al. 2026). By describing these patterns, we provide a baseline for where conservation effort should be placed.

## 2 Materials and Methods

### 2.1 Capture‐Mark‐Recapture Data of Arctic Terns in Denmark

The dataset we use here represents the combined effort of a group of Danish ornithologists who systematically ringed and measured adult and nestling terns in their nests at 242 breeding colonies (representing most of the breeding colonies of Arctic terns in Denmark) from 1931 to 2005 by capture. They also compiled previous and more recent ringing records and returns, that is, birds that had been ringed in Denmark and whose ring numbers were found or read at other ringing centres from dead or captured birds, reported to the Ringing Centre of the Zoological Museum, Danish Ornithological Central. Details on the dataset and its quality control can be found in Møller et al. (2006). We selected data with accurate coordinates and returned 15,015 records. We used this dataset to calculate the sampling effort which can partly reflect the size of each colony and to identify the natal and breeding dispersal events. Natal dispersal is defined as the relative change from the natal colony, and breeding dispersal as the relative change from the previous breeding colony; they can be confounded only if a juvenile makes a second dispersal before it is recaptured, but the chance of confounding is very low. All bird‐ringing activity in Denmark complied with local authorisation requirements for ringing and animal handling.

Of these, we identified 4410 recaptured individuals. Two thousand and six hundred and sixty‐six birds were ringed as adults at a breeding colony, then recaptured in at least one subsequent year. We identified 200 breeding (adult) dispersal events from those (captured from 1966 to 1985). A total of 1744 individuals were banded as chicks and later recaptured as breeding adults. We identified 890 natal (juvenile) dispersal events (captured from 1933 to 2000) from those. We used these data to calculate fidelity and observe dispersal patterns. The rest of the individuals were observed breeding at the same colony where they were initially banded.

While we did not observe secondary dispersal events among the young birds after natal dispersal, we recorded seven adults undertaking a second breeding dispersal and one undertaking a third.

### 2.2 Data Analysis

All data analyses were conducted using packages in R (R Core Team 2024). To explore the spatial clustering and distribution of colonies across Denmark, Kernel Density Analysis was conducted using the ‘ks’ package in R (Duong 2007). All breeding sites were treated as spatial points with equal weight. We selected the bandwidth using the plug‐in method (Hpi). The density surface was set on a 300 × 300 grid across the study area, and density values were scaled to represent the expected number of colonies per unit area. This method quantifies spatial aggregation by calculating the density of features within a specified neighbourhood around each output raster cell.

Dispersal distances were calculated as great‐circle distances (km) between departure and arrival coordinates for each recorded event using the ‘*geosphere*’ package (Hijmans 2010). Observations from the two datasets (natal dispersal and breeding dispersal) were combined while retaining group identity. Distance distributions were visualised using density‐scaled histograms to allow comparison between groups, both natal and breeding dispersal showed a right‐skewed distribution. The two distributions were compared using a two‐sample Kolmogorov–Smirnov test, which does not assume normality or equal variance between groups.

Temporal variation in dispersal activity was examined using annual summaries calculated separately for each dataset, including the number of dispersal events, sampling effort and the ratio of dispersal events to effort. These metrics were explored descriptively using time‐series plots.

Dispersal directions were derived as bearing angles between departure and arrival locations and treated as circular variables. Directions were grouped into equal angular bins spanning 0°–360° (where 0° = North) using the ‘*circular*’ package (Agostinelli and Lund 2025). Directional frequencies were visualised using stacked polar area plots. Directional data were further analysed using circular statistics. Mean directions were calculated for each dataset and temporal subset. Departure from circular uniformity was tested using Rayleigh's test. We also compared dispersal direction between natal and breeding dispersal using Watson's two‐sample test. To examine whether dispersal direction varied across time periods within each dispersal type, we used the Mardia–Watson–Wheeler test for natal and breeding dispersal across the three defined time periods (1967–1971, 1972–1976, 1977–1982) containing the majority of dispersal events.

A larger colony usually has more records. Since banding is performed on chicks and adults captured in the nest, the number of banding individuals in a colony is limited by the number of active nests; therefore, we used sample size as a proxy for community size. We examined the relationship between sampling effort and dispersal propensity by modelling how the site fidelity varied with the number of individuals sampled within each habitat. Analyses were conducted separately for breeding dispersal and natal dispersal datasets, with each observation characterised by a sample size and an associated site fidelity. To limit the influence of sparse data and ensure numerical stability, only sample groups with at least three observations were retained. The relationship between sample size and site fidelity was modelled using a generalised linear model (GLM) with a quasibinomial error distribution and a logit link function. Sample size was incorporated as a prior weight in the model. The model was specified as fidelity ~ sample, with weights = sample. Model predictions were generated, and 95% confidence intervals were calculated as ±1.96 standard errors. Observed data, fitted relationships and confidence bands were visualised using ‘ggplot2’ (Wickham 2016) to facilitate comparison.

To examine spatial connectivity, departure and arrival locations were discretised into grid cells by rounding coordinates to a fixed spatial resolution (0.1° × 0.1°). Cells within the study area were grouped using *k*‐means clustering (centres = 5) based on longitude and latitude, while cells outside the region were assigned to an auxiliary category. Directed movements between clusters were summarised using chord diagrams with the ‘circlize’ package (Gu et al. 2014), with self‐loops excluded. To compare within‐region or cross‐region dispersal, we classified the distribution of all colonies into five main regions with Arctic tern breeding colonies in Denmark following the *k*‐means clusters: Northern Jutland, Western Coast (separated into two subregions for natal dispersal), Copenhagen Metropolitan Area (Zealand), Funen (Fyn) and Storstrøm. Spatial patterns in dispersal origins were shown by a pie plot of dispersal choice for each region on the map. The proportion of within‐versus cross‐region movements was compared between natal and breeding dispersal using Fisher's exact test in R.

## 3 Results

### 3.1 Spatial Distribution of Breeding Colonies and Dispersal Choice of Different Regions

**FIGURE 1:**
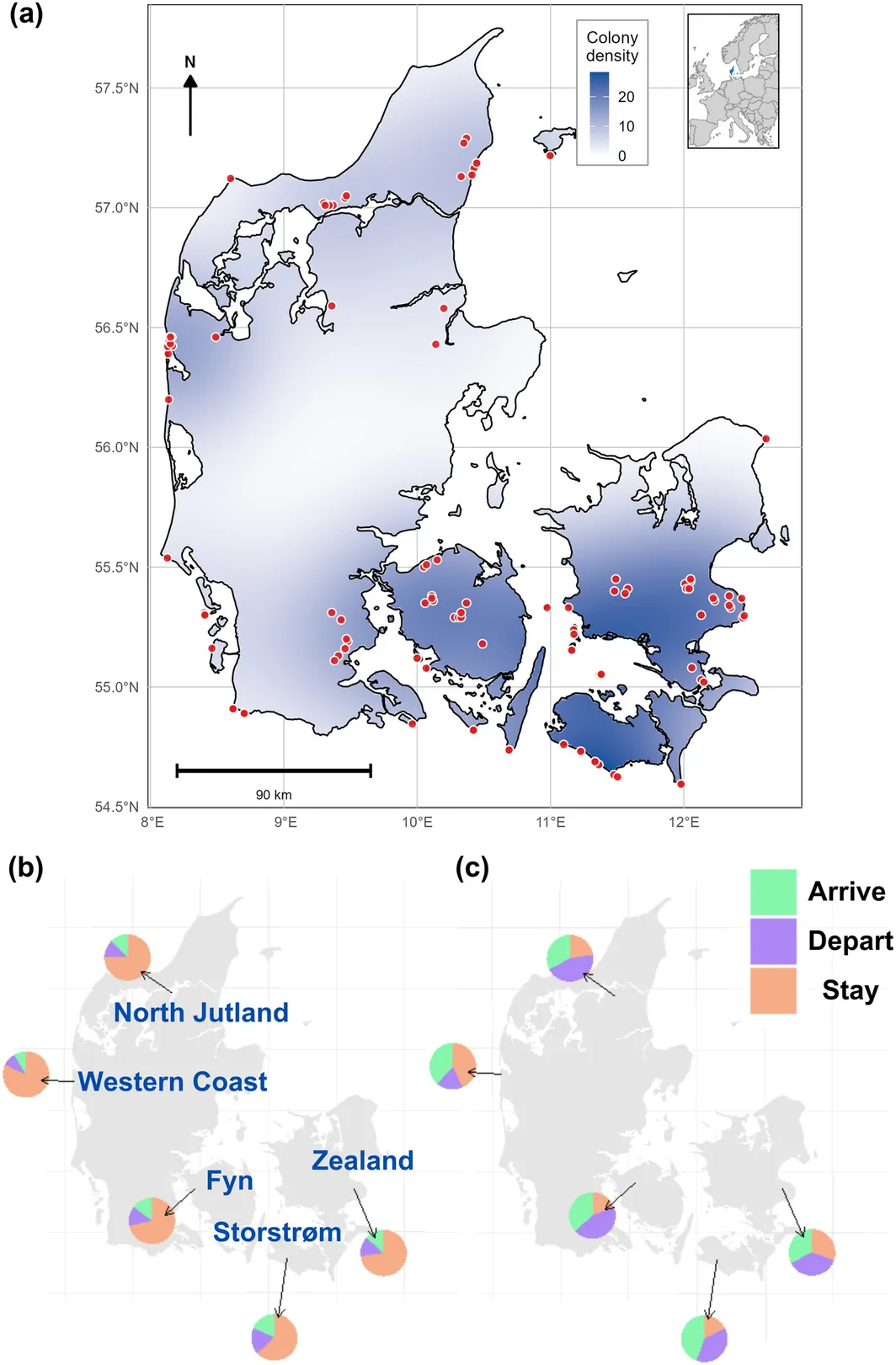
(a) Studied breeding colony density (per square km) of Arctic terns is unevenly distributed in Denmark; 0/white colour indicates absence of colonies in the area. Breeding dispersal (b) and natal dispersal (c) distribution by region. The pie diagrams show the proportion of recaptures at the same colony (in orange), recaptured elsewhere (in purple) or recaptured following immigration from another colony (in green).

Among the 242 breeding colonies included in this study, colonies were not evenly distributed along the coastline. We observed a distinct regional pattern of colony density, with greater densities recorded in the southeastern part of the country, exhibiting peak densities of 41.5 colonies per unit area on islands of Funen and Zealand (Figure 1a).

We found a higher proportion of juveniles (890 of 1744 recaptured individuals, 51.0%) leaving their primary colonies compared to adults (200 of 2666 recaptured individuals, 7.5%) in all regions (Figure 1b,c). Among recaptured adults, most of them returned to the same colony where they had been previously captured (Figure 1b). In contrast, recaptured juveniles were more likely to move to a different breeding colony for reproduction (Figure 1c).

### 3.2 Spatiotemporal Patterns of Dispersal Events

**FIGURE 2:**
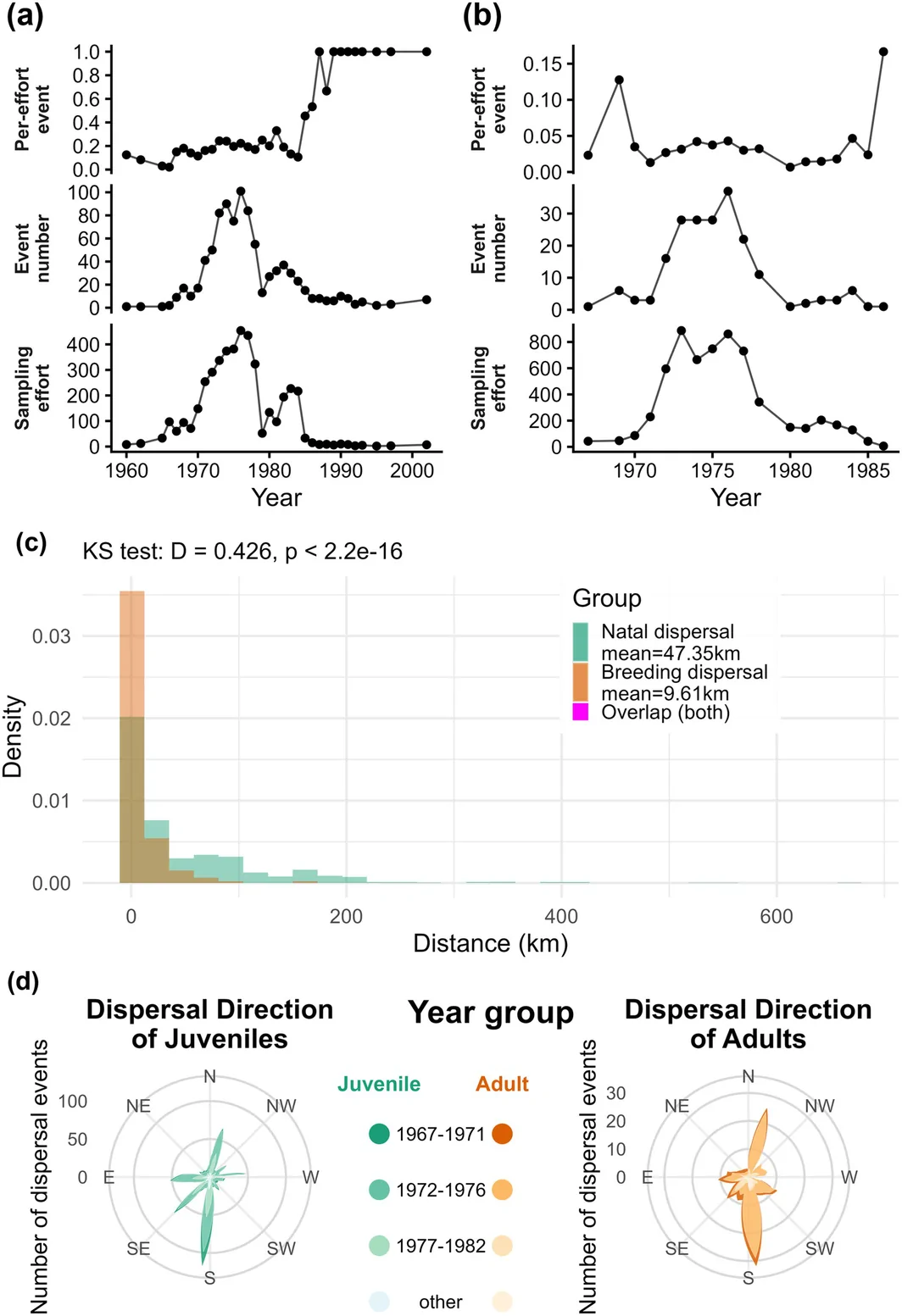
Dynamics and patterns of dispersal events. Annual variation in events per unit sampling effort, total number of dispersal events and sampling effort for juveniles (a) and adults (b) across the study period. (c) Kernel density distributions of dispersal distances for juveniles and adults, illustrating differences in the frequency of short‐ and long‐distance movements between age classes. (d) Circular distributions of dispersal directions for juveniles and adults, grouped by time periods.

Dispersal activity varied among years and differed between two types of dispersal (Figure 2a,b). For natal dispersal (U), events per unit effort were generally low in the early period (1960–1970) but increased in later years, which may be related to the bias of low data availability. Breeding dispersal (Ad) showed lower, more stable event rates per effort. The peak of events corresponds with sampling efforts in both age groups.

Dispersal distances differed significantly between natal and breeding dispersals (Figure 2c, two‐sample Kolmogorov–Smirnov test: *D* = 0.426, *p* < 2.2e‐16). Dispersal in both groups was dominated by short distances (natal: mean = 47.3 km, median = 16.0 km; breeding: mean = 9.6 km, median = 3.4 km). Dispersal directions further showed the difference between the two dispersal types and their temporal structure (Figure 2d). Both natal and breeding dispersals generally happened in a north‐west direction, with natal dispersals exhibiting relatively more occurrences in other directions. The dispersal direction was not random (average dispersal angle: natal: 236.3°, *p* = 0.0065; breeding: 249.97°, *p* = 0.0019). These mean directions did not differ significantly between the two dispersal types (Watson's two‐sample test: *U* ^2^ = 0.132, *p* > 0.10). Within each dispersal type, direction varied differently across the three time periods examined (1967–1971, 1972–1976, 1977–1982): natal dispersal direction shifted significantly across time periods from a westward mean direction in 1967–1971 (278.6°) to southwestward in 1972–1976 (233.1°) and west‐southwestward in 1977–1982 (251.8°) (Mardia–Watson–Wheeler test, *p* = 0.037), whereas breeding dispersal direction did not (*p* = 0.414).

**FIGURE 3:**
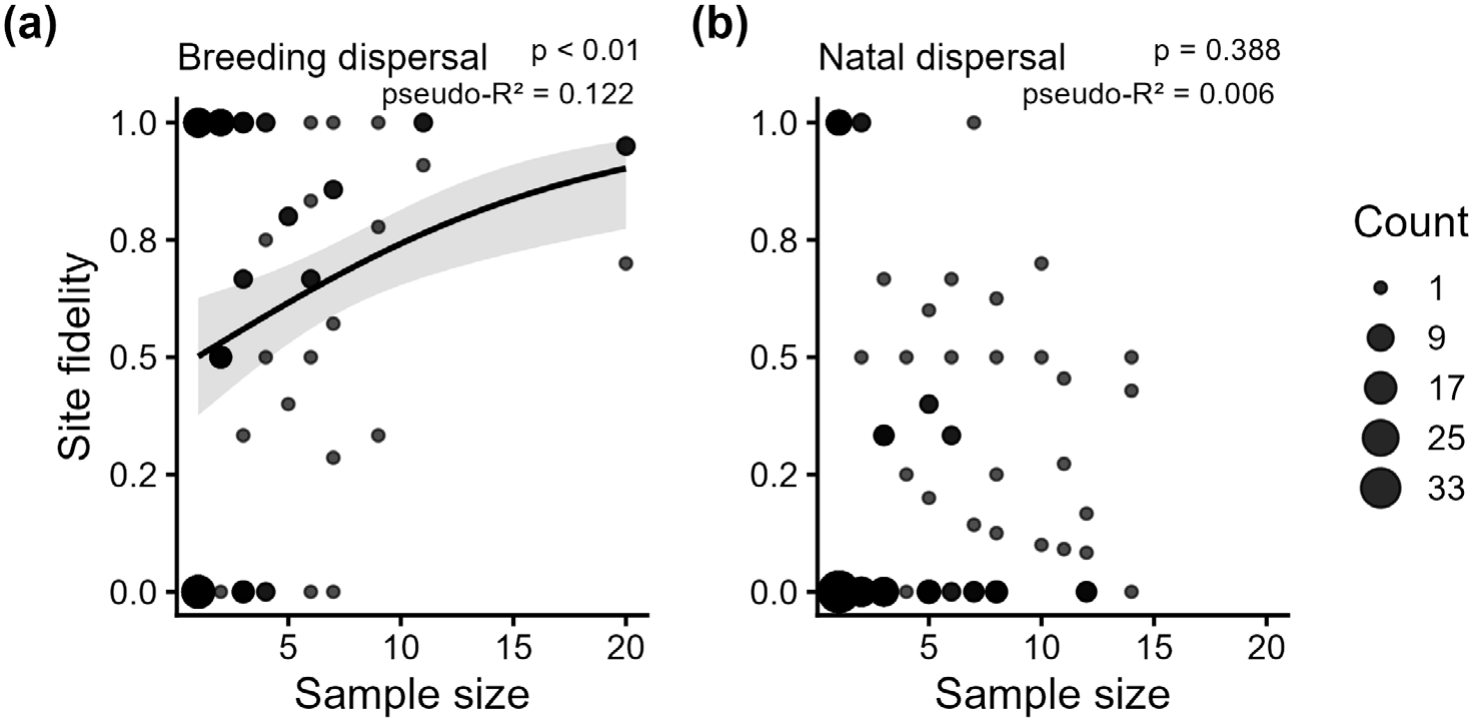
Effects of sample size on the fidelity of Arctic terns for adult (a) and juvenile (b). Fidelity data (0–1) were fitted against sample size, which is a proxy of colony population, using a generalised linear model (logistic regression). The model was weighted by sample size to adjust for heteroscedasticity among studies/sites with different sampling efforts.

Figure 3a,b shows the relationship between sample size and site fidelity, which means the proportion of individuals of known origin recaptured breeding at the same colony for both groups. For breeding dispersal (Figure 3a), the relationship indicates a positive trend, with the ratio of the individuals that breed in the same colony increasing as the sample size increases (slope = 0.117, SE = 0.035, *t* = 3.31, *p* = 0.001, *R* ^2^ = 0.122, *n* = 81 colonies). In contrast, the natal dispersal (Figure 3b) did not show a significant relationship between sample size and fidelity (slope = 0.034, SE = 0.039, *t* = 0.87, *p* = 0.388, *R* ^2^ = 0.006, *n* = 117 colonies). Overall, juvenile individuals showed greater flexibility in dispersal distance and direction, while adult individuals exhibited more conservative patterns.

### 3.3 Regional Patterns of Dispersal

**FIGURE 4:**
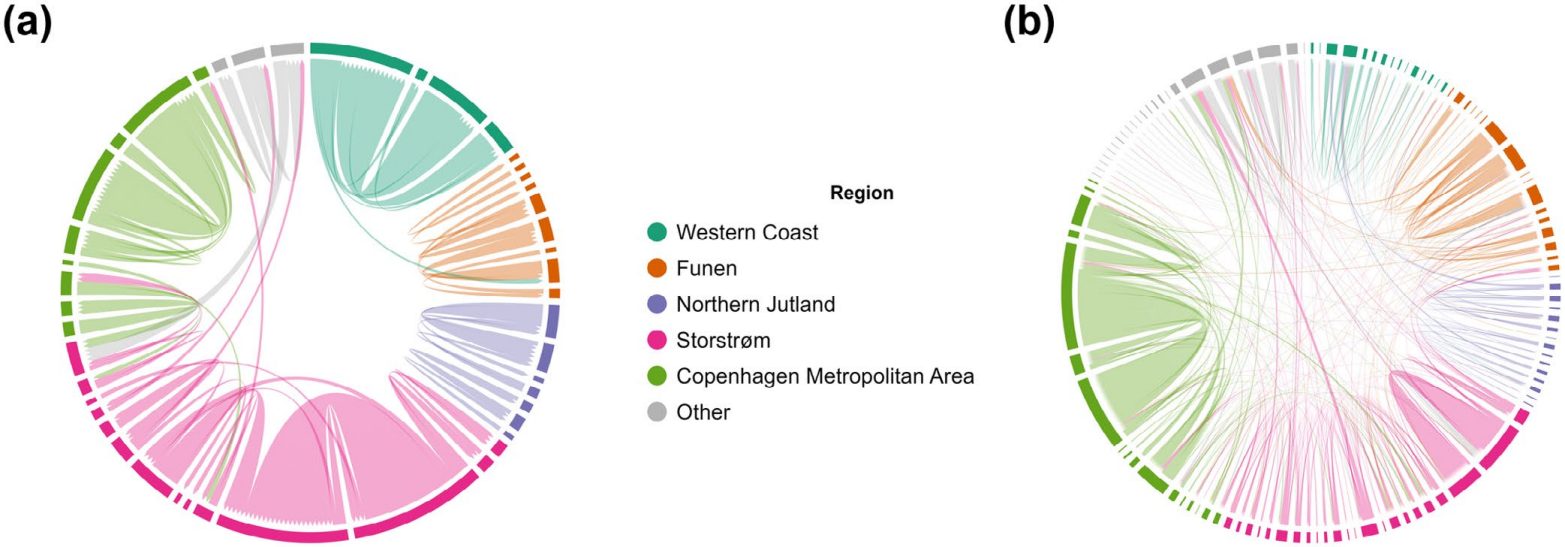
Chord plots show the connection between regions for breeding dispersal (a) and natal dispersal (b).

Chord diagrams highlighted pronounced differences in the spatial connectivity of dispersal between age classes. In breeding dispersal (Figure 4a), dispersal flows were strongly restricted within regions, with 112 of 120 dispersal events (93.3%) occurring within the same region. The network structure was dominated by intra‐regional or adjacent exchanges. In contrast, juvenile dispersal exhibited a more active spatial network (Figure 4b), with 495 of 629 classified movements (78.7%) occurring within the same region, and the remaining 134 (21.3%) across regions. This difference in within‐ versus cross‐region connectivity between the two dispersal types was statistically significant (Fisher's exact test: OR = 0.264, 95% CI [0.109, 0.557], *p* = 6.6 × 10^−5^), confirming that breeding dispersal is significantly more spatially restricted to within‐region movements than natal dispersal. In addition to within‐region movements, the natal dispersal generated more inter‐regional connections. These connections were distributed among a broader set of regional pairs, producing a more complex flow structure. As a result, the natal dispersal network displayed higher regional connectivity.

## 4 Discussion

This study revealed that the natal and breeding dispersal of Arctic terns differ in both scale and connectivity, with natal dispersal mostly acting as the primary driver of population‐wide mixing. Our study provides valuable information for targeted breeding site management based on specialised dispersal strategies for juveniles and adults, helping safeguard vulnerable seabird populations (Braby et al. 2012). Juveniles favour broader, more exploratory movement, and adults favour localised site fidelity, which might be a result of long‐term selection to maximise reproductive success under different life‐history constraints (Greenwood and Harvey 1982).

Our study showed that juveniles travelled longer distances, exhibited lower directional consistency and formed a highly interconnected movement network across Denmark and nearby countries (e.g., Germany, Sweden, Netherland). Together, these patterns suggest that first‐time breeders with less site knowledge and social constraints engage in broad‐scale exploration (Szostek et al. 2014; Redfern et al. 2026), consistent with the idea that natal dispersal contributes to gene flow and metapopulation persistence in long‐lived species (Sakao et al. 2023). In addition, this may also affect other evolutionary processes, such as avoiding inbreeding (Blomqvist et al. 2002) or competition with clutchmates and parents (Wakefield et al. 2017). Another possible mechanism might be related to the ‘prospecting’ hypothesis, which holds that first‐time breeders visit and assess multiple potential colonies before settling and ultimately choose a breeding colony better than the natal colony (Boulinier and Danchin 1997). However, to confirm this hypothesis, more behavioural studies on the postnatal and pre‐reproductive stages would be needed.

In contrast, adult breeding dispersal was even rarer and spatially constrained, reinforcing regional philopatry. Adults exhibited strong site fidelity, which increased with the size of the colony (GLM: slope = 0.117, *p* = 0.001; Figure 3a), with dispersal mostly occurring within regional clusters. These patterns might be explained by familiarity with local resources, social structure and previous breeding experience that may offset the potential benefits of relocation (Bialas et al. 2023), consistent with the view that breeding dispersal plays a limited role in habitat quality optimisation in this species (Redfern and Steel 2025; Chalfoun and Schmidt 2012). Site fidelity in long‐lived species is often favoured because it helps preserve pair bonds during breeding cycles, which are associated with higher breeding success (Sánchez‐Macouzet et al. 2014). Also, familiar neighbouring individuals can reduce the costs of territorial conflict and enhance group defence (Temeles 1994; Whittam and Leonard 2000). This relative behavioural inertia highlights the importance of existing colonies (Hauser 2001).

Our study has several limitations. First, our analyses rely on recaptured banding records, where the dispersal behaviour of individuals who were never recaptured, even those never banded, remains unknown. Thus, our estimates of dispersal proportion and site fidelity should be interpreted as conditional on recapture. Also, we did not resolve the wintering‐site situation, which might also influence breeding‐site choice due to data limitations (Egevang et al. 2010). With current advances in GPS and remote sensing technology, we could obtain more detailed data on bird movements and better understand this key process in long‐migratory birds. Future studies could compare present‐day dispersal rates and patterns with those described here.

Beyond the patterns described here, Arctic terns and other long‐migratory seabirds have faced a broader set of environmental pressures that make understanding dispersal increasingly urgent for conservation. In the context of the global expansion of the vehicle industry and increasing efforts to replace traditional fuels, the anticipated growth of the battery sector is likely to affect environmental emissions of heavy metals, including Hg, Cd and Pb (Harpprecht et al. 2024). At the same time, land use near breeding colonies has changed from semi‐cultivated agricultural to intensive agricultural in recent years, which is likely to lead to increased release of pesticides (European Environment Agency 2019; Levin 2022), fertilisers and noise from mechanical production, representing landscape‐level pressures (Newbold et al. 2015). Climate change can disrupt the synchronisation between breeding phenology and peak prey availability, potentially affecting both reproductive success and future dispersal decisions (Both et al. 2006). These risks could be much higher with the adults who exhibit high site fidelity for breeding, which constrains their capacity to leave colonies that are no longer suitable. In such a case, natal dispersal is more important for avoiding unfavourable breeding conditions. The protection of existing breeding habitats, as well as anticipating and establishing protected areas for potential new breeding habitats in advance, is essential for the conservation of Arctic terns.

The analysis of dispersal patterns described in this study is particularly relevant in the context of ongoing population declines and habitat loss affecting Arctic terns and other migratory seabirds. Over the past 30 years, Arctic tern populations in northwestern Greenland have experienced a marked and rapid decline (Burnham et al. 2017). Arctic migratory bird species may have lost 66%–83% of their former breeding habitats in recent years (Wauchope et al. 2017), intensifying selection pressures on remaining suitable sites. By quantifying typical dispersal and network connectivity patterns, this study delineates dispersal behaviour under recent historical conditions. Such a baseline may also provide a foundation for future monitoring, facilitating the detection of changes in dispersal distance or connectivity that may reflect emerging responses to increasing environmental pressures.

Nevertheless, conservation planning for long‐distance migratory birds remains constrained to individual protected areas, which is far from sufficient. Less than 10% of migratory bird species are adequately covered by protected areas throughout the entire annual cycle, while the majority spends most of their lives outside protected area boundaries (Runge et al. 2015; Marra et al. 2026). For a species migrating between polar regions, with its migration routes crossing multiple national jurisdictions and the high seas, protection secured at one node addresses only a part of the network; therefore, cross‐network coordination is essential (Morten et al. 2025, 2026). Furthermore, as bird breeding grounds redistribute through dispersal, conservation hotspots are not static, as their habitat requirements change over time and space (Runge et al. 2014). The dispersal distances and network structures quantified in this paper highlight this dynamic and underscore the importance of protecting all breeding grounds and potential future breeding grounds.

## 5 Conclusion

This study provides a detailed, long‐term description of natal and breeding dispersal patterns in Arctic terns across Denmark. The study should be of relevance for future research on dispersal patterns and population trends. Dispersal patterns described here provide a valuable reference point for predicting how to develop targeted conservation strategies for Arctic terns and other migratory seabirds responding to rapid environmental change.

## Author Contributions

**Haiyu Li:** conceptualization (equal), data curation (equal), formal analysis (equal), methodology (equal), visualization (equal), writing – original draft (equal). **Anders Pape Møller:** conceptualization (equal), data curation (equal), funding acquisition (equal), resources (equal). **Shengyu Wang:** validation (supporting), writing – review and editing (equal). **Céline Albert:** methodology (supporting), resources (supporting), writing – review and editing (equal). **Jacqui A. Shykoff:** conceptualization (equal), funding acquisition (equal), project administration (equal), supervision (equal), validation (equal), writing – review and editing (equal). **Ivan Jarić:** conceptualization (equal), funding acquisition (equal), project administration (equal), supervision (equal), validation (equal), writing – review and editing (equal).

## Funding

Funding for this research is provided by Université Paris‐Saclay.

## Conflicts of Interest

The authors declare no conflicts of interest.

## Data Availability

All relevant data and codes supporting this study are openly accessible at Dryad (https://10.5061/dryad.x69p8czxh).
